# The role of micro-organisms (*Staphylococcus aureus *and *Candida albicans*) in the pathogenesis of breast pain and infection in lactating women: study protocol

**DOI:** 10.1186/1471-2393-11-54

**Published:** 2011-07-22

**Authors:** Lisa H Amir, Meabh Cullinane, Suzanne M Garland, Sepehr N Tabrizi, Susan M Donath, Catherine M Bennett, Amanda R Cooklin, Jane RW Fisher, Matthew S Payne

**Affiliations:** 1 Mother & Child Health Research, La Trobe University, Melbourne, VIC 3000, Australia; 2 Women's Centre for Infectious Diseases, Bio 21 Institute, Parkville, VIC 3052, Australia; 3 University of Melbourne Department of Obstetrics and Gynaecology, The Royal Women's Hospital, Parkville, VIC 3052, Australia; 4 Murdoch Childrens Research Institute, The Royal Children's Hospital, Parkville, VIC 3052, Australia; 5 University of Melbourne Department of Paediatrics, The Royal Children's Hospital, Parkville, VIC 3052, Australia; 6 Deakin Population Health, Deakin University, Burwood, VIC 3125, Australia; 7 Parenting Research Centre, East Melbourne, VIC 3002, Australia; 8 Jean Hailes Research Unit, Monash University, Clayton, VIC 3168, Australia; 9 Centre for Women's Health, Gender and Society, University of Melbourne, Carlton, VIC 3053, Australia

## Abstract

**Background:**

The CASTLE (Candida and Staphylococcus Transmission: Longitudinal Evaluation) study will investigate the micro-organisms involved in the development of mastitis and "breast thrush" among breastfeeding women. To date, the organism(s) associated with the development of breast thrush have not been identified. The CASTLE study will also investigate the impact of physical health problems and breastfeeding problems on maternal psychological health in the early postpartum period.

**Methods/Design:**

The CASTLE study is a longitudinal descriptive study designed to investigate the role of *Staphylococcus *spp (species) and *Candida *spp in breast pain and infection among lactating women, and to describe the transmission dynamics of *S. aureus *and *Candida *spp between mother and infant. The relationship between breastfeeding and postpartum health problems as well as maternal psychological well-being is also being investigated. A prospective cohort of four hundred nulliparous women who are at least thirty six weeks gestation pregnant are being recruited from two hospitals in Melbourne, Australia (November 2009 to June 2011). At recruitment, nasal, nipple (both breasts) and vaginal swabs are taken and participants complete a questionnaire asking about previous known staphylococcal and candidal infections. Following the birth, participants are followed-up six times: in hospital and then at home weekly until four weeks postpartum. Participants complete a questionnaire at each time points to collect information about breastfeeding problems and postpartum health problems. Nasal and nipple swabs and breast milk samples are collected from the mother. Oral and nasal swabs are collected from the baby. A telephone interview is conducted at eight weeks postpartum to collect information about postpartum health problems and breastfeeding problems, such as mastitis and nipple and breast pain.

**Discussion:**

This study is the first longitudinal study of the role of both staphylococcal and candidal colonisation in breast infections and will help to resolve the current controversy about which is the primary organism in the condition known as breast thrush. This study will also document transmission dynamics of *S. aureus *and *Candida *spp between mother and infant. In addition, CASTLE will investigate the impact of common maternal physical health symptoms and the effect of breastfeeding problems on maternal psychological well-being.

## Background

The World Health Organization and Australian authorities recommend that babies are exclusively breastfed for the first six months of life [1,2]. During this time, and especially in the early weeks, breastfeeding women can experience a range of breastfeeding problems, in particular breast and nipple pain, or breast infections such as mastitis or "breast thrush".

Mastitis is an acute, debilitating infection that occurs in 15 to 20% of Australian breastfeeding women who experience a red, painful breast with fever [3,4]. Seventy five percent of mastitis episodes occur in the first seven weeks following birth [4]. It is a painful, distressing condition which may require hospitalisation or lead to development of a breast abscess [5]. Traditionally, *S. aureus *has been considered the most common aetiological agent of mastitis and is commonly isolated in infective mastitis and from breast abscesses [6]. However, recent studies have suggested that other micro-organisms such as coagulase negative staphylococci (CNS) may also play an important role in infectious mastitis [7,8]. CNS such as *Staphylococcus epidermidis *are normal inhabitants of the skin in healthy hosts and are considered commensal bacterial skin flora. The suggestion that CNS such as *S. epidermidis *may be involved in infective mastitis highlights that, although *S. aureus *is certainly one cause of mastitis, commensal skin flora which have previously been thought of as non-pathogenic, may also be responsible for this illness in a proportion of breastfeeding women [7].

Breastfeeding women may also experience burning nipple/breast pain known as breast thrush (not associated with breast redness or fever) which occurs in about 10% of breastfeeding women [9,10]. While the aetiology of mastitis has been investigated, the organism(s) associated with the development of breast thrush have not been identified. Some researchers and clinicians believe that the underlying pathogenesis in lactating women with burning nipple and breast pain is infection with *C. albicans *[10-12]. However, it has proved difficult to identify this organism from breast milk [13], and others attribute the pain to infection with *S. aureus *and treat women with long-term anti-staphylococcal antibiotics [14]. Another possibility is that this syndrome could be caused by multiple organisms. Co-infection with *S. aureus *and *C. albicans *or other *Candida *spp in the lactating nipple and breast may be leading to inflammation and pain. These organisms co-exist in infectious conditions in other moist parts of the body, such as angular cheilitis (in the corners of the mouth) [15,16] and paronychia (infection around the finger nail) [17]. This is currently a hotly debated issue and the pathogenesis of breast thrush is unclear [18,19].

In addition to breast symptoms and infection, maternal physical symptoms in the postpartum period are common. The Victorian Survey of Recent Mothers, a state-wide postal survey of a representative cohort of 1336 women, found that at six months postpartum, 94% of the sample reported one or more health problems [20]. The most common health problems reported were fatigue (69%), backache (43%), sexual problems (26%), haemorrhoids (25%) and perineal pain (21%) [20]. The impact of these postpartum health problems and breastfeeding problems on women's overall functioning and on their psychological well-being has not been widely investigated.

The CASTLE (Candida and Staphylococcus Transmission: Longitudinal Evaluation) study is a longitudinal study which aims to investigate the organisms involved in both breast thrush and mastitis. At recruitment, baseline samples will be collected from mothers' nipples, nose and vagina. Postpartum, we will collect follow-up samples from infants' mouths and nose, mothers' nipples and nose, and milk samples from each breast. We will also collect data on clinical outcomes, which will enable us to determine if *S. aureus *and/or *Candida *spp are involved in breast thrush. Furthermore, breast milk samples and nipple swabs will address whether coagulase negative staphylococci play a role in infective mastitis among participants. Data obtained will also allow us to describe the colonisation of infants with *S. aureus *and *Candida *spp, and investigate their transmission dynamics between mother and infant.

In addition to the microbiological aspects of the study, CASTLE will investigate the impact of breastfeeding problems and postpartum health problems on maternal psychological well-being. The repeated data collection intervals in the CASTLE study provide a unique opportunity to prospectively investigate the role breastfeeding difficulties and physical health complaints play in determining maternal mood and psychological well-being in the early postpartum period.

## Methods/Design

### Aims

Specific aims of the study are:

1. To determine whether *S. aureus *and/or *Candida *spp are involved in burning nipple/breast pain in lactating women - the condition known as breast thrush - using a longitudinal study for the first time;

2. To increase understanding of transmission dynamics and infant colonisation of these organisms by describing the timing of oral and nasal colonisation with *S. aureus *and *Candida *spp in breastfed infants and determining the role of infant colonisation with these organisms in relation to the timing of maternal mastitis and breast thrush;

3. To investigate comprehensively whether staphylococci other than *S. aureus *(coagulase-negative staphylococci) play a role in infective mastitis;

4. To ascertain the impact of common maternal physical health symptoms and breastfeeding problems on maternal psychological well-being at four and eight weeks postpartum, adjusting for known determinants of poorer postnatal mood.

### Study hypotheses

Pre-specified study hypotheses for the microbiological components of CASTLE are:

1. Women reporting nipple and breast candidiasis are more likely to have *Candida *spp isolated from their swabs and breast milk samples and/or from swabs taken from their infants;

2. Women reporting nipple and breast candidiasis are more likely to have *S. aureus *isolated from isolated from their swabs and breast milk samples and/or from swabs taken from their infants;

3. Participants experiencing mastitis are more likely to have *S. aureus *isolated from their breast milk compared to women not reporting this condition;

4. Infants of women reporting mastitis are more likely to have *S. aureus *isolated from their oral and/or nasal swabs compared to healthy controls;

5. Women who report an over-supply of breast milk are more likely to develop mastitis compared to women who report an adequate supply or under-supply of breast milk;

6. Women who report the use of hydrogel dressings in the postpartum period are more likely to develop mastitis compared to women who do not use these dressings;

7. Women who report a greater time between feeds are more likely to develop mastitis;

8. Women reporting nipple damage on the tip of their nipple are more likely to develop mastitis than women reporting no damage, or reporting damage elsewhere on the nipple;

9. Women administered antibiotics in labour or postpartum are more likely to develop nipple and breast candidiasis than women not treated with antibiotics during these periods.

Pre-specified study hypotheses for the psychological components of CASTLE are:

1. Women reporting two or more physical symptoms on at least two occasions between one and four weeks postpartum will have measurably worse psychological outcomes on two standardised assessments of maternal mood at four and eight weeks postpartum after adjusting for known determinants of poor maternal mood (lower antenatal mood, insufficient partner support, prior history of mood disturbance, personality style, and infant factors);

2. Women reporting breastfeeding problems (including mastitis, nipple/breast pain, low milk supply) will have measurably worse psychological outcomes on two standardised assessments of maternal mood at four and eight weeks postpartum after adjusting for known determinants of poor maternal mood (lower antenatal mood, insufficient partner support, prior history of mood disturbance, personality style, and infant factors);

3. Women reporting nipple or breast pain (> 5/10 on the pain scale) on two or more occasions, will have more psychological symptoms at the time of the nipple/breast pain than those experiencing less pain (≤ 5/10), or those reporting pain at only one study interval;

4. Women with mastitis will have more psychological symptoms at the data collection point at the time of the episode/immediately following the episode than those reporting no mastitis;

5. Women who have had breastfeeding problems resolved will have better mood scores than those unresolved at eight weeks;

6. Women continuing to breastfeed at eight weeks will have better mood scores than women not breastfeeding.

### Sample

Four hundred women and their newborn infants form the study group. Women are recruited in The Royal Women's Hospital (RWH), Melbourne and Frances Perry House (FPH), a private hospital located on the same site; approximately two hundred women from each site. Four hundred women will be recruited to allow for 20% loss as some may not breastfeed or give up prematurely/withdraw from study/lost to follow-up.

Eligibility criteria for the study group are: between 18 - 50 years of age; nulliparity; ≥ 36 weeks pregnant at recruitment; singleton pregnancy; breastfeeding intention for at least eight weeks postpartum; sufficient proficiency in English to complete written questionnaires and a telephone interview; residing ≤ 10 km from Melbourne Central Business District (CBD). Criteria for exclusion are: medical conditions which do not allow breastfeeding; breast reduction surgery; dermatitis on nipple during pregnancy; under care of the Women's Hospital Alcohol and Drug Service; under care of Mental Health Service or Social Worker.

### Sample size

Sample size estimations were conducted using Epi-Info Version 6. Table 1 shows power calculations for the study with alpha of 0.05 and beta of 0.20. Sample size calculations assume a ratio of 1:1 (unexposed: exposed) for comparison of infants colonised with *S. aureus *(assuming that about 50% of young infants are colonised with *S. aureus *[21]). To detect a clinically significant difference in the outcome of maternal breast thrush from 5% in the unexposed group to 15% in the group exposed to infant *S. aureus *colonisation, a sample of three hundred and eighteen women are needed. If we based the calculation on infants colonised with *Candida *spp (four not colonised to one colonised), two hundred and seventy women are required in order to have enough power to identify a clinically significant increase, a twofold relative risk in breast thrush in mothers of infants with oral *Candida *spp (20% to 40%). Therefore, a sample size of four hundred was considered sufficient to detect clinically important differences in breast thrush and mastitis associated with infant colonisation with *S. aureus *and *Candida *spp, (alpha = 0.05, beta = 0.20), allowing for 20% loss to follow-up or withdrawal from the study. This will allow us to examine other outcomes of interest, including determinants of maternal mastitis.

**Table 1:** Sample size estimations for the CASTLE study

Exposure ratio (unexposed: exposed)	Outcome of interest	Difference	Sample size estimate
**Infant no nasal *S. aureus */infant nasal *S. aureus *(1:1)**	Mother breast thrush	5% versus 15%	318
**Infant no nasal *S. aureus */infant nasal *S. aureus *(1:1)**	Mother mastitis	12% versus 25%	308

**Infant no oral Candida/infant oral Candida (4:1)**	Mother breast thrush	20% versus 50%	130
**Infant no oral Candida/infant oral Candida (4:1)**	Mother breast thrush	20% versus 40%	270

### Recruitment procedures

Participants are recruited antenatally by one of two methods. Research assistants speak at antenatal breastfeeding classes run by both RWH and FPH to introduce the study. Interested women attending these classes provide a contact telephone number and are contacted when they are thirty six weeks pregnant and recruited to the study after giving informed consent. Alternatively, participants are recruited at routine antenatal visits at either the public antenatal outpatient clinics (RWH) or at private obstetric practices (FPH), after at least thirty six weeks of pregnancy. Potential participants are approached in the clinic waiting areas, and their English competency, parity and intention to breastfeed ascertained.

If interested in the study, verbal and written information about the study is offered. Eligible women are given an invitation package which contains: (i) a leaflet explaining the purpose of the study and inviting women to take part; (ii) a plain language statement with further detail about the study and study consent forms; (iii) a sheet for recording contact details and, (iv) the first study questionnaire (Q1). Signed consent and participant contact details are obtained. At recruitment, baseline samples are collected from mothers' nipples, nose and vagina.

### Follow-up procedures

All contact with study participants after recruitment is managed by the CASTLE research team based at Mother & Child Health Research (MCHR). Researchers visit the postnatal wards each day to identify whether any participant has given birth in the previous twenty four hours. Participants who are in the hospital following birth are visited by the researcher in the first day(s) after birth, and are invited to complete a written questionnaire. Samples are collected from infants' mouths and nose and mothers' nipples and nose. A sample of colostrum is collected from each breast if possible.

At the end of weeks 1, 2, 3 and 4 postpartum, researchers visit participants at home to collect the same samples for the microbiological aspects of the CASTLE study. During these home visits, participants again complete written questionnaires. If participants cease breastfeeding during this time, no further home visits are conducted. At week 8, regardless of breastfeeding status, participants are contacted by telephone, to complete a final questionnaire.

### Ethical issues

Each participant gives informed consent to participate in the study and for information to be obtained from their hospital medical records, with the condition that they are free to withdraw at any time. Participants can consent to the microbiological aspects and the psychological aspects of the study separately.

Upon withdrawal, participants can request that data arising from their participation are not used in the research project provided that this right is exercised within four weeks of the completion of their participation in the project.

Any information obtained in connection with this project, and which could identify participants will remain confidential and be stored in a secure location at MCHR, La Trobe University. Only the chief investigator, study co-ordinator and research assistants see personal information.

Participants are assigned a unique study number and all data associated with the study are identified using this number. Data will be stored in a securely locked cabinet at MCHR for five years after the date of publication and then destroyed in a secure manner.

Ethics approval for the study has been granted by:

• La Trobe University Human Ethics Committee (06-078);

• RWH Human Research Ethics Committee (06/41 for the microbiological aspects of the study and 09/48 for the psychological components of the study);

• Human Research Ethics Committee of the University of Melbourne (1033949);

• Medical Advisory Committee at FPH.

### Data collection

#### 1. Questionnaires

At recruitment, women complete a questionnaire to collect information about maternal age, gestational age, intended length of breastfeeding duration, history of thrush and staphylococcal infections, history of allergies (asthma, hay fever, eczema and dermatitis) [22], antibiotic use, highest level of educational attainment, and marital status. Participants recruited to the psychological component of CASTLE also complete a written self-report questionnaire comprised of the Profile of Mood States (PoMS) [23], the Depression, Anxiety and Stress Scale (DASS) [24] and the Vulnerable Personality Scale (VPS) [25]. Prior history of psychological problems is also ascertained.

Follow-up questionnaires at weeks 1, 2, 3, 4 and 8 postpartum collect information about nipple/breast pain, milk supply, breast candidiasis, mastitis, nipple damage, other infections and antibiotic use. Information on the use of breast pumps and milk expression is also obtained [26]. The birthing method, induction of labour, administration of antibiotics during labour and perineal trauma are also collected in the first postpartum questionnaire.

Women are asked if they are fully breastfeeding (i.e. no infant formula), if the baby is receiving expressed breast milk, if the baby is receiving formula and whether the majority of feeds are breast milk or formula. There is no standard way of diagnosing mastitis. Therefore, we ask about a range of breast symptoms and associated fever or flu-like symptoms as has been used previously [3,27]. The definition of mastitis is the development of at least two breast symptoms (pain, redness, lump) and at least one systemic symptom (fever or flu-like symptoms) [3,27]. Francis-Morrill and colleagues [28] found that nipple pain, associated with a shiny or flaky appearance of the nipple and breast pain had a high positive predictive value for nipple candidiasis. In this study we are asking about burning nipple pain, stabbing breast pain and asking participants if they have been given a diagnosis of nipple/breast candidiasis by any of their clinicians. Data on treatments used are also collected. Researchers are documenting nipple appearance and any nipple damage, as described in previous studies [29,30] and breasts are assessed for evidence of spreading infection. An algorithm will be designed to enable a likely diagnosis on nipple and breast candidiasis which incorporates women's reported symptoms, clinician's diagnosis of nipple or breast thrush and clinical appearance.

At weeks 1, 2, and 3 weeks postpartum, participants completing the psychological components also complete a short questionnaire on physical health symptoms and the global self-rated assessment of physical health from the SF-36 [31]. Participants also complete two visual analogue scales (VAS) to assess "depression" and "emotional lability", developed by Kendell et al. [32]. At week 4 and 8, participants complete the PoMS and DASS [23,24] in addition to the above (Table 2).

**Table 2:** Summary of psychological component of the CASTLE study

Study interval	Breast symptoms (collected in CASTLE study)	Physical symptoms	SF-36* self-rated physical health	DASS-21**	PoMS^†^	Visual analogue scale, maternal mood^#^
Recruitment				•	•	

Hospital	•					

Week 1	•	•	•			•

Week 2	•	•	•			•

Week 3	•	•	•			•

Week 4	•	•	•	•	•	•

Week 8	•	•	•	•	•	

*SF-36 self-rated global physical health item [31]

**Depression, Anxiety and Stress Scales (DASS-21) [24]

^†^Profile of Mood States (PoMS) [23]

#"Depression" and "Lability" items [32]

Infant behaviour and infant health are assessed using a six-item scale rating infant sleeping, feeding and settling and maternal experiences in caring for their infant. These items have been adapted from those used by investigator colleagues in a community cohort study of nine hundred postpartum women [33]. Respondents are asked to indicate any health problems or illnesses that the infant has experienced, and when these episodes occurred. Support from their intimate partner is also assessed. Two items will assess support from partner since the birth, including the adequacy of practical support they have received from their partner since the birth, and whether or not they can confide in their partner.

#### 2. Microbiology

Samples are collected at each visit with participants (Table 3) and are labelled with the date of collection, participants' date of birth and an identification number unique to each participant. Mothers provide nasal, nipple (both nipples) and vaginal swabs and breast milk samples (both breasts). Two nipple swabs are obtained from each nipple: a standard charcoal swab for microbiological analysis (Copan Diagnostics Inc.) and a flocked swab for molecular analysis (Copan Diagnostics Inc.). Nasal and oral swabs are obtained from infants. Baseline nasal, nipple and vaginal swabs are obtained at recruitment. Follow-up nasal, nipple and milk samples are taken from women; follow-up nasal and oral swabs are obtained from infants. A vaginal swab is obtained when women present prior to birth if possible.

**Table 3:** Schedule of specimen collection for the CASTLE study (400 women)

		RECRUITMENT	LABOUR	HOSPITAL	WK 1	WK 2	WK 3	WK 4
**Mother**	**Nasal**	1		1	1	1	1	1

	**Vaginal**	1	1					

	**Left nipple**	2*		2*	2*	2*	2*	2*

	**Right nipple**	2*		2*	2*	2*	2*	2*

	**Left breast milk**			1	1	1	1	1

	**Right breast milk**			1	1	1	1	1

								

**Baby**	**Nasal**			1	1	1	1	1

	**Oral**			1	1	1	1	1

	**TOTAL**	**6**	**1**	**9**	**9**	**9**	**9**	**9**

* 2 swabs taken, one for culture analysis and one for molecular analysis

Nasal swabs are used for culture of *S. aureus*. Nipple (charcoal), oral and vaginal swabs are used for culture of *S. aureus *and *Candida *spp. Breast milk samples are used for culture of *S. aureus*, CNS and *Candida *spp and nipple swabs (flocked) are used for molecular identification of *Candida *spp. The identity of tentative *S. aureus *isolated from all samples is subsequently confirmed using molecular methods which also analyse isolates for methicillin resistance. CNS isolated from breast milk samples are being stored to identify individual species and strains by molecular techniques. Further specimens are obtained from participants where appropriate (e.g. swab of a caesarean wound infection) and used for culture of *S. aureus *and *Candida *spp.

In addition, all women are given a 70 ml sterile container (Sarstedt Australia Pty. Ltd.) to be used to collect a sample of milk should they develop mastitis during the first eight weeks postpartum. They are asked to express some milk (1 - 2 ml) from the affected breast into the jar and contact one of the research assistants to organise collection of the sample. This milk sample is analysed as previously described.

With the exception of the vaginal swab, samples are collected by the research assistant unless a participant specifies a wish to collect the samples. Hands are sanitised and clean disposable gloves worn when collecting samples. Hands are re-sanitised and a fresh pair of gloves is worn for the collection of each subsequent sample. Nasal and nipple swabs are moistened in sterile saline (Thermo Fisher Scientific, Australia Pty. Ltd.) prior to sample collection. Nasal swabs are rotated four times around the inside of the right nasal vestibule and then placed in Amies charcoal transport media for analysis. Both the standard and flocked nipple swabs are rolled over the nipple and areola together using a ten-point swabbing technique [34] paying particular attention to any cracks/fissures which may be present. Vaginal swabs are taken from approximately 1 cm inside the vagina. Oral swabs are taken from the dorsum of the tongue. All standard swabs are placed in Amies charcoal transport media prior to analysis.

After nipple swabs have been collected, the nipple/areola region is washed twice using sterile water wipes (Briemar Nominees Pty. Ltd.). The research assistant then expresses and discards the first drops of breast milk (approx 500 μl) before collecting mid-stream milk (1 - 2 ml) into a sterile container. All samples are transported in an insulated container at 4°C to the Bacteriology Laboratory at the Royal Children's Hospital within 1 - 2 hr of collection. Samples remain refrigerated until analysed.

*S. aureus *growth is indentified using Vogel-Johnson (VJ) agar [35]. This bacterium possesses the ability to reduce tellurite to metallic tellurium, which results in growth as black colonies. In addition, *S. aureus *is able to ferment mannitol, resulting in a distinct yellow halo around the black colony. During the initial 24 hr of incubation contaminating organisms are almost completely inhibited by tellurite, lithium chloride and the high glycine concentration of the medium. All tentative *S. aureus *are then sub-cultured onto Mannitol Salt (MSA) agar (Thermo Fisher Scientific, Australia Pty. Ltd.) and pure cultures stored in glycerol broths at -80°C.

MacConkey (MAC) agar (Thermo Fisher Scientific, Australia Pty. Ltd.) is used to identify the presence of CNS. Samples are plated onto MAC plates and morphologically distinct isolates present are streaked onto MSA and incubated for 24 hr. Due to the high salt concentration of this medium, growth of most bacterial species except staphylococci and micrococci are inhibited. Colonies that are able to ferment mannitol produce a distinct yellow colour change in the media and are tentative *S. aureus *isolates. *S. aureus *and CNS specimens are stored in glycerol broths at -80°C for further molecular typing.

Candida CHROMagar is used to identify candida to the species level of *C. albicans, C. glabrata, C. krusei *and *C. kefyr *[36]. All fungal isolates (including those other than the four previously mentioned species) are stored in glycerol broths at -80°C.

#### 3. Molecular analysis

Like many fungal genera, it is difficult to sensitively detect *Candida *spp by conventional culture techniques. This is especially true for samples such as swabs and non-blood fluids, including breast milk [37-40]. To increase sensitivity for the detection of *Candida *spp from the nipple, a flocked swab is resuspended in 1× Phosphate Buffered Saline (PBS) and DNA extracted from this is subsequently used in a polymerase chain reaction (PCR) which has been used previously to successfully identify *Candida *spp [41,42].

Collected nipple swabs are analysed by quantitative, real-time PCR. Nucleic acid is extracted from samples using the Siemens Versant kPCR automated extraction platform according to the manufacturer's recommendations (Siemens Ltd.). A 10 μL aliquot of the resulting DNA from two samples (left and right nipples) of each woman is screened using Taqman probes specific for *C. albicans, C. krusei *and *C glabrata*. A broad-range, *Candida *spp probe is also included in the multiplex assay. These probes target the RNase P RNA gene RPR1 [43], and are highly specific for their target sequences.

Confirmation of tentative *S. aureus *isolates and screening for methicillin resistance is carried out using multiplex PCR targeting the *nuc *gene of *S. aureus *and the *mecA *gene of methicillin-resistant *S. aureus *(MRSA) [44]. We plan to carry out pulsed-field gel electrophoresis (PFGE) on selected isolates of *S. aureus*. This will be carried out at the Gram-Positive Bacteria Typing and Research Unit, Perth, and will allow comparison with globally relevant community and clinical strains. The PFGE profiles from *S. aureus *identified from mothers (nasal, nipples, milk and vagina) will be compared to isolates cultured from her infant's mouth to ascertain whether the same strain of the organism is present and to determine transmission dynamics.

Species level identification of tentative CNS isolates is determined by PCR targeting the *Staphylococcus *spp *tuf *gene, which encodes for the prokaryotic elongation factor TU [45]. Following CNS isolate confirmation, isolates are stored for subsequent molecular typing. We plan to genotype CNS isolates using random amplified polymorphic DNA (RAPD) analysis [46]. The profiles of isolates from an equal number of milk samples of women with mastitis and without mastitis will be compared to see if there is a correlation between particular strains and breast infection.

### Data analysis plan

The study co-ordinator monitors specimen collection and ensures results are complete. Data from the questionnaires is entered into EpiData by an experienced research assistant, and transferred to Stata for analysis. The laboratory presents the microbiological results in an Excel spreadsheet with is also imported into Stata for analysis.

Descriptive analysis includes maternal demographic characteristics, details about the birth, about breastfeeding duration, nipple pain, breastfeeding difficulties, mastitis and maternal physical symptoms. Maternal and infant characteristics and transmission dynamics are described.

The relationship between infant colonisation with *S. aureus *and infant colonisation with *Candida *spp and maternal breast thrush will be tested by chi-square (or Fisher's exact if more appropriate). We will model the timing of incidence of maternal mastitis using survival analysis, with maternal and infant *S. aureus *carriage as time-dependent co-variates, together with appropriate variables (e.g. maternal age, method of birth, nipple damage). We will model the presence of *Candida *spp in vaginal/infant oral or nipple/milk samples and a diagnosis of nipple/breast candidiasis.

For the psychological components of the study, the primary outcomes are the two standardised assessments of maternal psychological symptoms - the PoMS and the DASS [23,24]. Multivariate analyses (linear regression) will be used to ascertain how much of the variation in maternal psychological symptoms can be accounted for by the presence of physical problems and breastfeeding difficulties on the primary outcomes of maternal well-being (DASS, PoMS) at four and eight weeks postpartum. Known risk factors for worse maternal mood will be adjusted for including unsettled infant behaviour, insufficient partner support, vulnerable personality traits and previous history of mood disturbance. Pregnancy mood will also be adjusted for. Cross-sectional comparisons of maternal mood will be conducted for women experiencing nipple/breast pain and/or mastitis and those without nipple/breast symptoms.

## Discussion

### Changes to the protocol after the commencement of recruitment in November 2009

Initial Ethics approval for the study had been granted by the La Trobe University Human Research Ethics Committee and the RWH Human Research Ethics Committee for the microbiological aspects of the study, and recruitment began in November 2009. Ethics approval for the maternal physical and mental wellbeing component of CASTLE was obtained on 2 June 2010. Women were then invited to take part in the full study, but could consent to the microbiological aspect of CASTLE independently of the psychological component of the study. We had recruited ninety eight women to CASTLE before the introduction of the psychological aspects of the study (Table 4).

**Table 4:** Implementation and timeline for the CASTLE study

Date	Activity
May 2009	Study co-ordinator appointed to oversee all aspects of CASTLE

Sept 2009	Approval granted from Medical Advisory committee at Frances Perry House (7 September 2009). Approval already granted by La Trobe University (2 November 2008) and the Royal Women's Hospital (20 February 2007). Tracking database set-up. Questionnaires, protocols and procedures finalised.

Oct 2009	2 part-time (0.5) research assistants appointed

Nov 2009 - June 2010	Research scientist commences. Recruitment of participants to the microbiological aspects of the study and follow up for 8 weeks.

June 2010 - May 2011	Approval for psychological component of CASTLE granted by the Royal Women's Hospital (2 June 2010). Recruitment of participants to the microbiological and psychological aspects of the study and follow up for 8 weeks.

Feb 2010 - Sept 2011	Data entry and cleaning

Sept 2011 - March 2012	Data analysis

Sept 2011 - Sept 2012	Summary of results to participants Write up papers for publication Presentation at conferences

Our original aim had been to recruit twenty two women each month over an eighteen month period. However, we were not achieving this and therefore implemented several strategies in August 2010 aiming to increase our recruitment figures to twenty eight women per month (Table 4). The strategies included (i) increasing the presence of research assistants at Pregnancy Clinics at RWH; (ii) employment of additional research staff to carry out postnatal visits and an additional scientist to increase efficiency of microbiological analysis; (iii) increasing the recruitment zone from ≤ 10 km to ≤ 16 km from Melbourne CBD.

### Strengths and limitations of the study

The CASTLE study is a prospective longitudinal study which will collect data and microbiological samples from participants over time from approximately thirty six weeks of pregnancy gestation until eight weeks postpartum. This enables us to study transmission dynamics of *S. aureus *and *Candida *spp, as well as investigate the natural history of mastitis and breast thrush. A study of this detail investigating the role of *S. aureus*, *Candida *spp and CNS in the pathogenesis of breast pain and infection in lactating women has not been conducted previously. We need to understand these causal pathways before we can develop interventions to reduce these infections in new mothers. Previous breastfeeding studies have been cross-sectional; microbiological studies, while longitudinal, have not collected clinical data. Furthermore, the repeated data collection intervals in the CASTLE study provide a unique opportunity to prospectively investigate the role breastfeeding difficulties and physical health complaints play in determining maternal mood and psychological well-being in the early postpartum period.

One limitation of the CASTLE study is that participants are not representative of Australian women. Only nulliparous women able to read and write in English, who have reached thirty six weeks of pregnancy, planning to breastfeed for at least eight weeks after birth and who live ≤ 16 km from the Melbourne CBD are eligible to participate in the study. The rationale for restricting eligibility to women who have reached thirty six weeks gestation was to limit the number of preterm births in the study as these infants may suffer health complications and are less likely to breastfeed successfully. The reasoning behind restricting participation to nulliparous women was to more accurately follow the transmission dynamics of *S. aureus*, CNS and *Candida *spp from mother to baby without previous breastfeeding history influencing maternal colonisation. Because of the number of home visits to be completed by research assistants involved in the study, we originally decided on a recruitment limit of ≤ 10 km from the Melbourne CBD. However, to increase recruitment figures, we increased this zone to ≤ 16 km from the CBD in August 2010.

In addition, since approximately one third of Australian women have private health insurance, this study has an over-representation of women with private insurance. These women may have an intention to breastfeed for longer and tend to be more willing to participate in an interview study. However, as the main aim is to describe the pathogenesis of nipple/breast candidiasis, it was important to recruit women with a high expectation of breastfeeding for at least eight weeks as well as be willing to complete participation.

## Abbreviations

CASTLE: Candida and Staphylococcus Transmission: Longitudinal Evaluation; CBD: Central business district; CNS: Coagulase-negative staphylococci; DASS: Depression, Anxiety and Stress Scale; DNA: Deoxyribonucleic acid; FPH: Frances Perry House; MAC: MacConkey agar; MCHR: Mother & Child Health Research; MRSA: Methicillin-resistant *S. Aureus*; MSA: Mannitol salt agar; PBS: Phosphate buffered saline; PCR: Polymerase chain reaction; PFGE: Pulsed-field gel electrophoresis; PoMS: Profile of Mood States; RAPD: Random amplified polymorphic DNA; RWH: Royal Women's Hospital; SF-36: Short Form 36, Health status measure; VAS: Visual analogue scale; VJ: Vogel-Johnson agar; VPS Vulnerable Personality Scale

## Competing interests

The authors declare that they have no competing interests.

## Authors' contributions

LHA conceived the original idea for the study. All authors have contributed to the development of the study. MC is study co-ordinator. MSP is conducting the microbiological and molecular analysis. All authors read and approved the final manuscript.

## Pre-publication history

The pre-publication history for this paper can be accessed here:

http://www.biomedcentral.com/1471-2393/11/54/prepub
